# A case report of sigmoid colon cancer with the inferior mesenteric artery directly originating from the superior mesenteric artery

**DOI:** 10.1186/s40792-023-01671-2

**Published:** 2023-05-24

**Authors:** Kiyotaka Mizoguchi, Kinuko Nagayoshi, Yusuke Mizuuchi, Koji Tamura, Masafumi Sada, Kohei Nakata, Kenoki Ouchida, Masafumi Nakamura

**Affiliations:** Department of Surgery and Oncology, Graduate School of Medical Sciences, Kyushu University, 3-1-1 Maidashi, Higashi-ku, Fukuoka, 812-8582 Japan

**Keywords:** IMA anomaly, Sigmoid colon cancer, Laparoscopic low anterior resection

## Abstract

**Background:**

There are few reports describing the unusual origin of the inferior mesenteric artery (IMA). We report a rare case of advanced sigmoid colon cancer with the IMA arising from the superior mesenteric artery.

**Case presentation:**

A 59-year-old man with diarrhea and abdominal distention was diagnosed with advanced sigmoid colon cancer. Colonoscopy revealed a semi-circumferential cancer lesion in the sigmoid colon. Enhanced CT scan and CT angiography showed that the IMA directly originated from the superior mesenteric artery at the level of the second lumbar vertebra. PET-CT suggested metastases in the para-intestinal lymph nodes and the liver, but not in the central lymph nodes along the IMA. Preoperative diagnosis was sigmoid colon cancer cT4aN2aM1a cStage IVA(UICC, 8th edition). We performed laparoscopic complete resection as the radical treatment of the primary region prior to resection of the liver metastases. Intraoperative findings showed that the IMA was running parallel to the abdominal aorta; meanwhile, the colonic autonomic nerve was supplied from the lumbar splanchnic nerve at the caudal side of the duodenum. Central lymph nodes around the colonic autonomic nerve were dissected en bloc with the regional lymph nodes. Pathological radical resection including the regional lymph nodes metastasis was achieved. Two months later, complete resection of the liver metastasis was performed. After the adjuvant chemotherapy, no recurrence was observed 1.5 years after the liver resection was performed.

**Conclusions:**

Preoperative confirmation of the anatomy helped us to safely complete radical surgery in a patient with unusual bifurcation of the IMA.

## Background

Three major arteries arise from the abdominal aorta: the celiac artery, the superior mesenteric artery (SMA), and the inferior mesenteric artery (IMA). While there are many various subtypes of the branches from the celiac artery or the superior mesenteric artery reported, there are few reports of those from the IMA [1, 2]. Here, we report a case of advanced sigmoid colon cancer with the IMA arising from the SMA.

## Case presentation

**Fig. 1:**
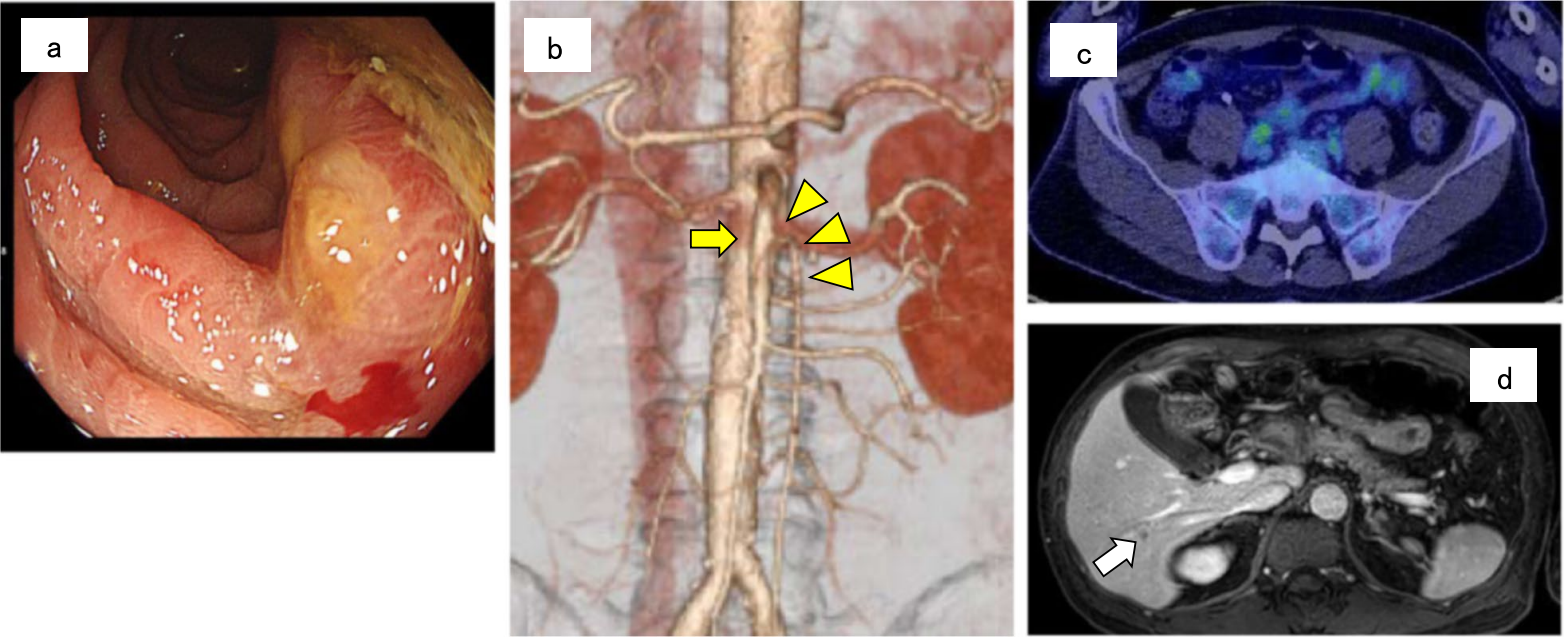
Preoperative imaging findings. **a** Colonoscopy showed a semi-circumscribed irregular elevated lesion in the sigmoid colon. **b** 3D-CT angiography showed that the IMA (yellow arrowheads) originated from the SMA (yellow arrow). **c** On PET-CT, there was no abnormal accumulation in the central lymph nodes around the IMA. **d** MRI showed findings suggestive of liver metastasis (white arrow)

A 59-year-old-male presented with a complaint of bloody stools. On physical examination, no mass was felt during palpation. Laboratory data on admission showed that the levels of tumor markers including CEA and CA19-9 were within normal ranges. Colonoscopy revealed a semi-circumscribed irregular elevated lesion in the sigmoid colon that was diagnosed as adenocarcinoma by biopsy (Fig. 1a). Abdominal enhanced CT scan revealed wall thickening of the sigmoid colon, and 3D-CT angiography showed that the IMA originated at 36 mm distal side from the SMA, which was the same level as the second lumbar vertebra, and the first jejunal artery bifurcated further distal side from the IMA origin (Fig. 1b). It also detected that the middle colic artery branched off from the SMA at 27 mm distal side from the IMA origin, and both the left colic artery and the sigmoid colic artery bifurcated from the IMA. No direct branches originating from the abdominal aorta were identified to supply the left-side colon. There were no anomalies or abnormalities detected except for the IMA. On PET-CT, paracolic lymph nodes were suspected of metastasis, but there was no abnormal FDG accumulation in the lymph nodes around the root of the IMA (Fig. 1c). Liver metastasis was also suspected by PET-CT and MRI (Fig. 1d). The patient was diagnosed with sigmoid colon cancer cT4aN2aM1a cStage IVA. Therefore, we planned treatment with laparoscopic low anterior resection followed by resection of the liver metastases to achieve radical resection.

**Fig. 2:**
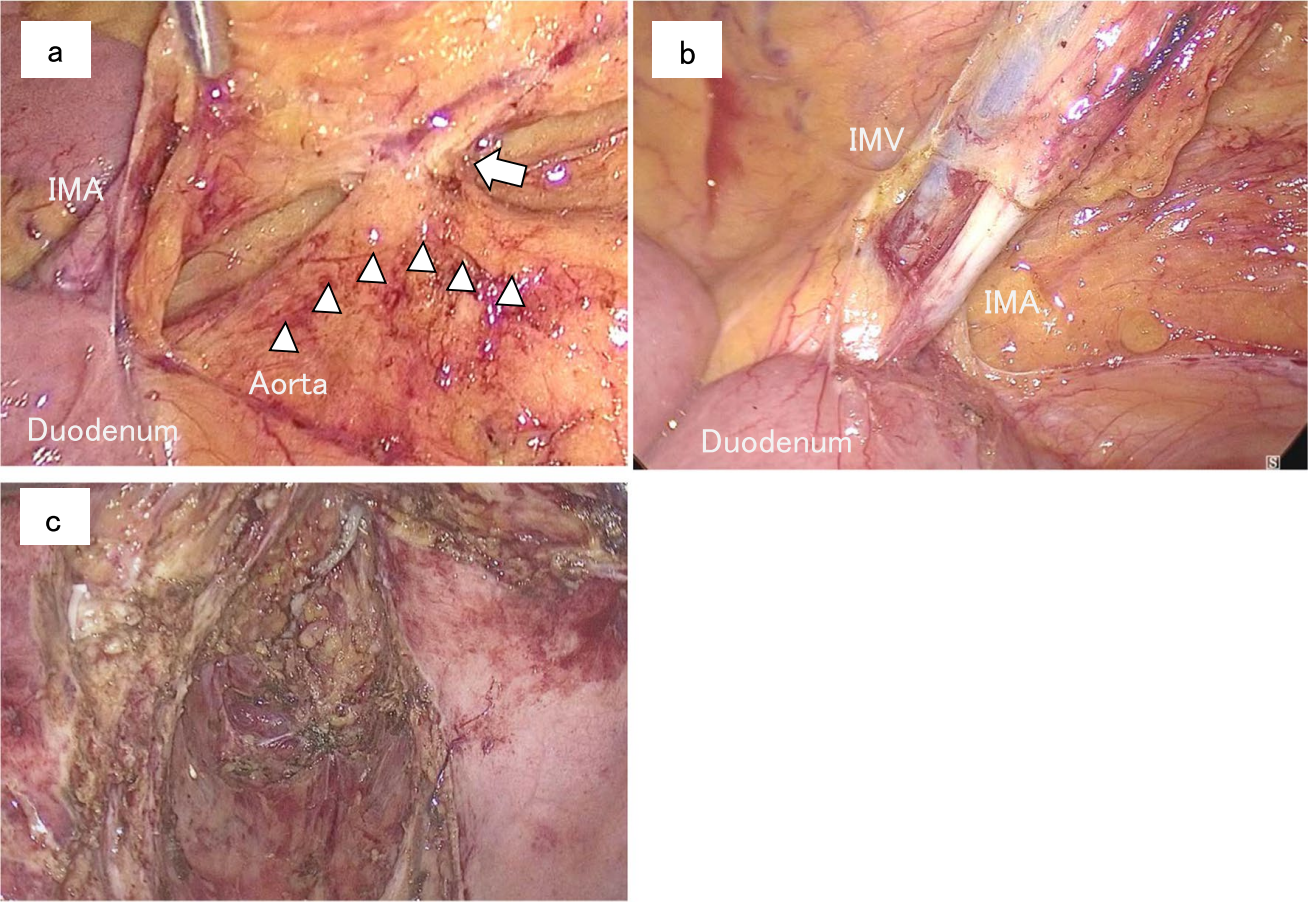
Intraoperative findings during laparoscopic low anterior resection. **a** The IMA ran parallel to the abdominal aorta without branching, while the neural branch (white arrow) arising from the lumbar splanchnic nerves (white arrowheads) supplied the left-sided colon. **b** The central lymph nodes around the IMA were dissected, followed by transection of the IMA at the level of the inferior edge of the duodenum. **c** Complete resection was performed

Laparoscopically, we can found that the tumor was located in the sigmoid colon and involved the small intestine and the left seminal vesicle. The IMA was identified as running parallel to the abdominal aorta; meanwhile, the colonic autonomic nerve was supplied from the lumbar splanchnic nerve at the caudal side of the duodenum (Fig. 2a). Central lymph nodes around the colonic autonomic nerve were dissected en bloc with the regional lymph nodes, followed by resection of the IMA at the level of the lower duodenal border (Fig. 2b). The left seminal vesicle, the left pelvic plexus and the middle rectum were firmly adhered to the tumor, and thus we performed low anterior resection with combined resection of them (Fig. 2c). Indocyanine green fluorescence system was used for assessment of the blood supply to the colonic stump. Pathological radical resection including the regional lymph nodes metastases was achieved. Two months later, complete resection of the liver metastasis was performed. After adjuvant chemotherapy, no recurrence was observed during the 1.5 years since the liver resection was performed.

## Discussion

Anatomical variation of the IMA is relatively rare. Kakihara et al. reported that, among 3,182 cases of abdominal angiography, one case (0.03%) had a defective IMA, two cases (0.06%) had abnormal running of the root associated with visceral retroversion, and two cases (0.06%) branched from the SMA. All other cases bifurcated to the left side at the level of the third lumbar vertebra [3]. Several primitive mesenteric arteries arise from the dorsal aorta in a segmental fashion and are distributed against their respective primaries in the early fetal period. Early in development, there is a longitudinal anastomosis connecting the arteries vertically in the dorsal mesentery. According to fetal development, some primitive mesenteric arteries disappear, while the others become the celiac artery, the SMA, and the IMA, which are distributed in the foregut, midgut, and hindgut, respectively [4]. The IMA is initially formed at the twelfth thoracic vertebra but eventually migrates to the level of the third lumbar vertebra [5]. In this case, the IMA bifurcation from the SMA may occur due to both abnormal arterial disappearance and lack of the usual migration. According to the previous reports, the IMA arising from the SMA typically diverges as the first branch supplying the colon (as shown in Table 1) [2, 3, 6–16]. In most cases, the left colic artery arises as a branch of the IMA. One-third of the previous cases were associated with other vascular malformations. In the present case, the IMA was the first branch of the SMA and had the same bifurcation pattern as most previous cases, which included the left colic artery and the sigmoid colic artery. This case also showed a branch from the lumber splanchnic nerve supplying the left-sided colon at the level of the third lumber vertebra where the IMA normally branches. It is possible that the sympathetic nervous system and the vascular system develop asynchronously.

**Table 1:** Summary of previous cases of the IMA arising from the SMA

Author	Year	Age	Sex	Order of the IMA branching from the SMA origin	LCA branching from the IMA	Associated arterial anomalies
Gwyn DG	1966	76	male	2nd	Present	No other abnormalities
Kitamura S	1987	69	male	1st	Present	No other abnormalities
Yamasaki M	1990	61	female	1st	Absent*	Anomaly of the middle colic artery
Kakihara N	2001	66	male	1st	Present	Hepatomesenteric trunk
Osawa T	2004	79	male	1st	Not described	Hepatomesenteric trunk
Yi S-Q	2008	79	male	1st	Present	Gastrophrenic trunk, hapatosplenic trunk
Maleux G	2010	64	female	1st	Not described	Accessory mesenteric artery to the ileum
Yoo SJ	2011	82	female	2nd	Present	No other abnormalities
Okuda	2019	63	male	1st	Present	No other abnormalities
Kondou H	2015	63	male	1st	Present	No other abnormalities
Minamisawa K	2019	66	female	1st	Present	No other abnormalities
Matsutani Y	2020	75	male	1st	Present	No other abnormalities
Korai T	2021	65	female	1st	Absent (described as aMCA)	No other abnormalities
The present case	2023	59	male	1st	Present	No other abnormalities

*IMA* inferior mesenteric artery, *SMA* superior mesenteric artery, *LCA* left colic artery

*arising from a common trunk with right and middle colic arteries

For left-sided colorectal cancer with unusual IMA bifurcation, the optimal definition of the extent of lymph node dissection has not been established [3, 13–16]. Kakihara et al. performed radical lymph node dissection including around the root of the IMA branching from the SMA for cT3N0M0 cStage II lower rectal cancer [3], while Kondo et al. performed central lymph node dissection up to the level of the inferior edge of the duodenum where the IMA usually branches from the aorta, preserving blood flow to the LCA for rectal cancer cT3N0M0 cStage II [14]. Minamisawa et al. dissected the lymph nodes around the bifurcation of the LCA as the cranial border of the dissection for cT2N0M0 cStage I rectal cancer [15]. In this case, preoperative PET-CT suggested metastases in the para-intestinal lymph nodes, but not in the proximal lymph nodes along the IMA. Pathological examination revealed complete resection; therefore, central lymph node dissection including that at the level of the inferior border of the duodenum would be oncologically sufficient. When the IMA was deficient, the marginal artery from the middle colic artery was the only blood flow to the left-sided colon. Thus, regional lymph node dissection in the direction of the intestinal axis is more important than central lymph node dissection in such cases.

Although anatomical variation of the IMA is infrequent, preoperative assessment using CT and CT angiography is very important because they can provide clear case-specific anatomical information. Even in patients with rare anatomical anomalies, prior confirmation of the anatomy allows us to safely complete radical surgery.

## Conclusions

We reported a rare case of sigmoid colon cancer with the IMA directly branching from the SMA. Preoperative confirmation of the anatomy enabled us to safely complete radical surgery even in a patient who had unusual bifurcation of the IMA.

## Data Availability

Not applicable.

## Abbreviations

CA: Celiac artery
SMA: Superior mesenteric artery
IMA: Inferior mesenteric artery
CT: Computed tomography
PET-CT: Positron emission tomography-CT
CEA: Carcinoembryonic antigen
CA19-9: Carbohydrate antigen 19-9
MRI: Magnetic resonance imaging
LCA: Left colic artery
